# Electrochemical Sensing in 3D Cell Culture Models: New Tools for Developing Better Cancer Diagnostics and Treatments

**DOI:** 10.3390/cancers13061381

**Published:** 2021-03-18

**Authors:** Micaela Oliveira, Pedro Conceição, Krishna Kant, Alar Ainla, Lorena Diéguez

**Affiliations:** 1 Medical Devices Research Group, International Iberian Nanotechnology Laboratory (INL), 4715-330 Braga, Portugal; micaela.oliveira@inl.int (M.O.); pm.conceicao@ua.pt (P.C.); krishna.kant@inl.int (K.K.); alar.ainla@inl.int (A.A.); 2 Chemistry Department, University of Aveiro, 3810-193 Aveiro, Portugal

**Keywords:** cancer diagnostics, electrochemical biosensing, tissue culture system, precision diagnostics, 3D cell culture

## Abstract

**Simple Summary:**

Over the last few years, there has been a scientific revolution with the appearance of organ-on-a-chip models that overcome the limitations of conventional 2D systems, while reproducing more faithfully the in vivo features of tissues and organs. The integration of sensors in these systems allows the monitoring of a variety of parameters that could be relevant for the study of diseases. Electrochemical biosensors are ideal candidates for this integration, since they can be miniaturised and are very reliable in real-time continuous measurements of a large panoply of relevant biomarkers. In the context of cancer, these electrochemical cancer-on-a-chip models have the potential to become essential tools for the study of cancer development and drug efficacy.

**Abstract:**

Currently, conventional pre-clinical in vitro studies are primarily based on two-dimensional (2D) cell culture models, which are usually limited in mimicking the real three-dimensional (3D) physiological conditions, cell heterogeneity, cell to cell interaction, and extracellular matrix (ECM) present in living tissues. Traditionally, animal models are used to mimic the 3D environment of tissues and organs, but they suffer from high costs, are time consuming, bring up ethical concerns, and still present many differences when compared to the human body. The applications of microfluidic-based 3D cell culture models are advantageous and useful as they include 3D multicellular model systems (MCMS). These models have demonstrated potential to simulate the in vivo 3D microenvironment with relatively low cost and high throughput. The incorporation of monitoring capabilities in the MCMS has also been explored to evaluate in real time biophysical and chemical parameters of the system, for example temperature, oxygen, pH, and metabolites. Electrochemical sensing is considered as one of the most sensitive and commercially adapted technologies for bio-sensing applications. Amalgamation of electrochemical biosensing with cell culture in microfluidic devices with improved sensitivity and performance are the future of 3D systems. Particularly in cancer, such models with integrated sensing capabilities can be crucial to assess the multiple parameters involved in tumour formation, proliferation, and invasion. In this review, we are focusing on existing 3D cell culture systems with integrated electrochemical sensing for potential applications in cancer models to advance diagnosis and treatment. We discuss their design, sensing principle, and application in the biomedical area to understand the potential relevance of miniaturized electrochemical hybrid systems for the next generation of diagnostic platforms for precision medicine.

## 1. Introduction

2D cell culture is the dominant methodology for cell culture assays. However, the spatial limitations prevent an accurate representation of living tissues and distort their proper functioning [1]. Throughout the years, different 3D culture techniques have emerged, in order to establish a bridge between two dimensional models and in vivo models. From transwell plates, spheroids, microcarriers, and scaffold-based models, to more complex systems, such as organoids or microfluidic chips, 3D culture has become increasingly a more desirable alternative to the traditional 2D in vitro models [2].

Like biological tissues require blood vessels to carry nutrients, waste, and exchange chemical signals, advanced in vitro models also need a well-controlled mass transport around the cells. This is achieved with microfluidics, which is technology handling small volumes of fluids (10^−9^ to 10^−18^ L) across microchannels with dimensions from ten to hundreds of micrometers [3]. Microfluidic chips can also incorporate sensors to monitor the system. One of the most common and readily integrated sensing approaches for microfluidic device is based on electrochemistry, which has become an integral part of cell biology studies, for applications in drug development/screening, cell sorting, and tissue engineering, among others. The adaptability of microfluidics embraces great potential for several applications in the cell biology and biomedicine fields. These microfluidic devices can be used to generate the so-called organs-on-chip (OoCs), also referred to as MPS (Micro Physiological Systems). An OoC is a microsystem with chambers at the macro scale, where living cells can grow and are continuously perfused [4]. These systems mimic the physiological environment of organs by introducing key parameters such as concentration gradients, mechanical compression, fluid shear-force, and by allowing organized cell patterning [5]. These miniaturized systems have been developed for several organs and tissues, including lung [6], liver [7], gut [8], kidney [9], blood-vessels [10], and metastatic tissue as well [11].

In the last 20 years, cancer research has been directed towards using 3D culture alternatives, as 2D cultures commonly provide misleading data, for instance, related to drug efficacy. Tumorigenesis is known to be controlled by the microenvironment where the cells are embedded [12], which cannot be properly replicated in 2D models. Biochemical and mechanical signaling arising from the extracellular matrix (ECM) influences the tumour progression, through the binding of soluble molecules, the recognition of peptic motifs in ECM components by cellular adhesion receptors, and even the sensing of matrix stiffness [12]. The study of metastasis has also progressed by the introduction of microfluidic Metastasis-on-chip (MoC) models that can be personalized and can help predict the outcome of treatments for each patient. In a nutshell, the adequate model can help to identify the key mechanisms involved in tumour evolution, and consequently, develop new therapeutic strategies or improve the existing ones [12].

Along with the advancements in the field of OoC, there is a need to introduce tools to keep monitoring the viability and metabolic activity of the tissue. Monitoring provides information about the cellular state and metabolism. The need for monitoring of cell metabolites, for example, can be driven by different objectives, whether the main concern is to gain a better understanding of metabolic pathways or to assess drug response [13]. In cancer research, sensing tools are pivotal to monitor tumour evolution and for drug screening.

Monitoring techniques range from standard microscopy for cell imaging to integrating sensors that measure physical, chemical, and biological parameters [14,15]. Despite ongoing advances in 3D cell culture, there is still limited literature that describes new systems with integrated sensors [16,17] and hence plenty of opportunities to integrate new sensors in OoCs. Though optical microscopy based on fluorescence (e.g., epifluorescence or confocal) is still the most common approach to monitor cellular activity in OoCs [18], it is closely followed by electrochemical sensing. This is due to the ease of integration, and miniaturization of the electrodes, making the applicability very wide [19]. Compared with optical sensors, electrochemical sensing is very attractive due to high sensitivity, fast response times, experimental simplicity, integration capacity, and low cost, as electrochemical devices do not require large equipment or high-end instrumentation to transform chemical information into an electrical signal [20,21,22]. Moreover, when compared with optical measurements, electrochemistry offers some advantages such as being label-free and not being affected by the matrix interferences [23].

Integrated electrochemical sensors can provide on-line monitoring of many relevant parameters [16,24] and can be easily miniaturised to be used in small settings, with low power and small reaction volumes, with high sensitivity, even though sensitivity and limits of detection of such sensors are more limited [20,25]. Lactate production, glucose consumption, and reactive species concentration (ROS and RNS) have been reported consistently as parameters determined through electrochemical methods. Nevertheless, electrochemical sensing is not delimited to the monitoring of quantitative metabolic parameters. Sorting and detection of cancer cells have also been described in the literature, as potential applications for the use of electrochemical tools [26,27].

The scope of this review is to compile recent progresses in the development of new cell culture models with integrated electrochemical sensing and their applications. Obstacles for the future advances of 3D cell culture models with integrated sensing features are also discussed.

## 2. From 2D to 3D Cell Culture in Cancer

Cell culture systems and animal models are crucial to study the formation of organs and tissues, and their function in healthy and pathological conditions. Nowadays, these cell culture systems are enabling the biotechnology industry to produce proteins and vaccines, and to prepare cell-based assays for drug screening [17,28]. Traditional in vitro and in vivo models have been instrumental in understanding human cell growth and differentiation in a laboratory setting, while mimicking the real in vivo microenvironments [2]. However, for the proper understanding of the mechanisms of disease using these systems, it is important to monitor 3D morphology and protein production, among other various important aspects [29,30]. To promote cell growth and to obtain reliable results, these systems need to replicate the tissue/tumour microenvironment and supply cells with essential nutrients, including amino acids, carbohydrates, vitamins, minerals, and growth factors. Current 2D and 3D cell culture models are normally grown in a petri dish, a multi-well plate attached to a solid or semi-solid substrate (monolayer or adherent culture), in flasks (suspension culture) or lately inside microfluidic devices. All these cell cultures are usually monitored by using optical microscopy. However, integrating electrochemical sensing methods adds further possibilities for efficient continuous control of the microenvironment [31].

### 2.1. Limitations of 2D Cell Culture

**Figure 1:**
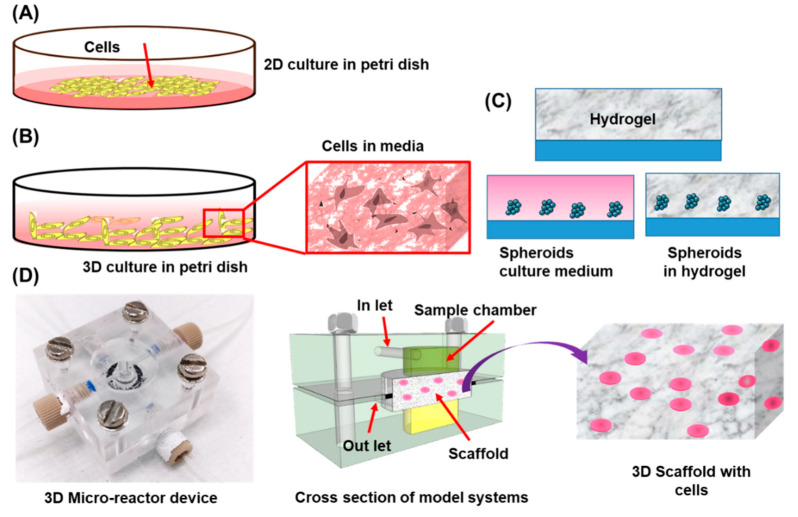
Schematic representation of (**A**) 2D cell culture in a petri dish and (**B**) 3D cell culture in a petri dish using a hydrogel. (**C**) Spheroid culture in hydrogel matrices (**D**) 3D cell culture in a small dynamic microreactor containing a hydrogel matrix in the middle of the two chambers.

Traditional monolayer cell cultures use surfaces (dishes, flasks or wellplates) as mechanical support for 2D cell growth (Figure 1A). Some cells can also grow in suspension culture [32]. In these monolayer cultures, the cells have access to nutrients and gases through the medium, which they also use to regulate their physical and chemical environment [33,34]. Since the medium is limited in standard arrangements, it needs to be changed every couple of days. Small changes in the culture process are usually not recorded and hence key information in the cell microenvironment remains unexplored.

Electrochemical sensors can be easily incorporated into 2D or 3D cell microenvironments to monitor changes such as pH or cell growth [23]. Jochen Kieninger et al. developed an innovative approach for sensing cells in a 2D culture Flask (SCCF) using microfabricated electrochemical sensors. The integrated sensors were incorporated in the flask surface to allow uninterrupted cell growth, while the transparent wall of flask allowed optical inspection in real time during measurements. Multiple experiments were performed in parallel flasks. The model system was applied for the study of brain tumour and breast cancer, using these amperometric sensors to monitor oxygen levels in a variety of culture conditions. The pH of the media was also monitored using potentiometric sensors based on iridium oxide. This system offered a ground for development of sophisticated cell monitoring systems using electrochemistry [35]. In spite of various advances in 2D cell culture methodologies, the cells move freely in culture plates, which could have an impact on the success of electrochemical measurements. In addition, the 2D cell cultures do not mimic the in vivo microenvironment of real tissues, where cells are surrounded by an extracellular matrix (ECM) [32].

### 2.2. 3D Cell Culture

Recent developments in 3D cell culture systems allow cells to grow with the aid of synthetic or natural ECMs or scaffolds mimicking the 3D structure of tissues and organs. These 3D systems create artificial microenvironments allowing cells to grow onto a 3D support structure (Figure 1B) [36]. 3D culture systems can be based on the use of scaffolds that promote cell proliferation, migration, and aggregation (Figure 1D) [29]. Non-scaffold-based 3D cell culture systems rely on the formation of cell aggregates or spheroids that create their own ECM (Figure 1C) [37]. Normally, these 3D systems can be fabricated following either of these two options: ‘’bottom-up’’ and ‘’top-down’’. In the ‘’bottom-up’’ approach, single cells or spheroids are used to build complex tissue structures. On the other hand, ‘’top-down’’ cell cultures grow under predefined shapes and sizes following the scaffold structure [38].

### 2.3. Relevance of 3D Models in Cancer

There is a pressing need to have robust 3D cell culture systems that properly mimic diseased and healthy tissue, while incorporating sensing capabilities to monitor cellular processes and to quantify small molecules in the extracellular environment. Scientists in cancer research have extensively proved that the microenvironment is responsible for developing mechanisms of drug resistance. 3D cell culture has been proposed for drug screening, not only due to ethical issues of animal testing, but also because of the expensiveness of animal models. Moreover, the literature has proven how different drugs responses can be in 2D and 3D cell cultures [39]. The very presence of an ECM matrix linking cells together affects cell responses to drugs by altering their action mechanism and their resistance. The use of 3D model systems can also help to understand the mechanisms of cell migration and aid in the detection of whole cells in the circulation of cancer patients. Circulating tumour cells (CTCs) are tumour cells originating from the solid tumour that travel around the body through blood circulation, with the capacity to cause metastasis. These CTCs have demonstrated a strong potential towards early diagnosis of cancer and selection of personalised treatment. Nowadays, CTC detection and isolation has become a hot research field with detection technologies based on microfluidic systems. The isolation of cancer cells remains a challenge because of their low concentration in blood samples. Therefore, reliable detection of CTCs requires extremely sensitive and specific analytical tools [40]. CellSearch™ system is the only CTC detection system approved by the FDA in the market to provide prognostic information in metastatic breast, prostate, and colorectal cancers [41].

## 3. Electrochemical Techniques for Cell Monitoring

**Figure 2:**
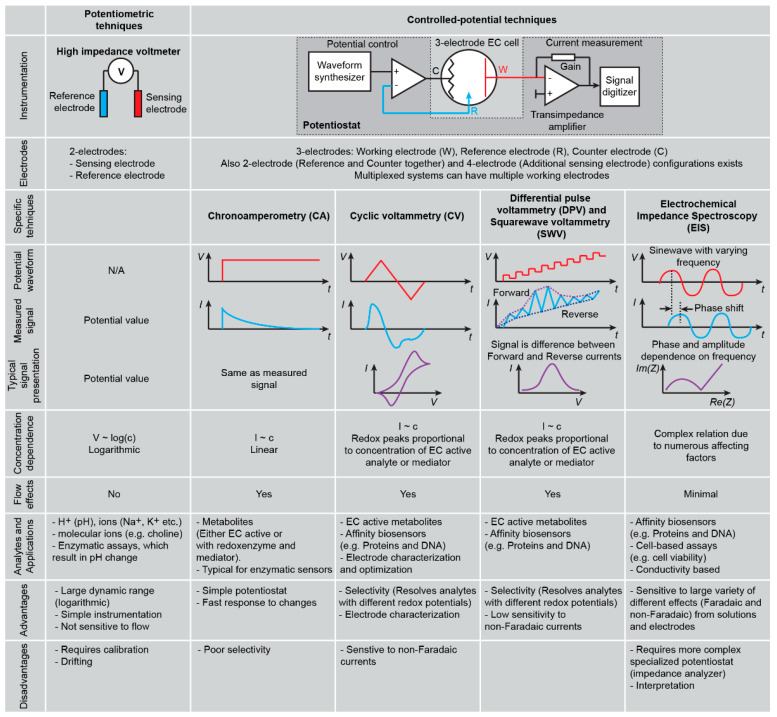
Summary of the main electrochemical techniques used in cell cultures and organ chip (Abbreviations: EC electrochemistry, N/A—not applicable, H^+^ hydrogen, Na^+^ sodium, K^+^ potassium, DNA Deoxyribonucleic acid).

Electroanalytical chemistry provides a cornucopia of techniques for characterization of biological samples and there are four main reasons making it particularly attractive for on-chip cell culture systems: (i) electrochemical electrodes can be readily miniaturized down to the size of even single-cells [42,43], (ii) electrodes can be microfabricated and easily integrated with microfluidic platforms [44], (iii) electrochemical sensors are cost-effective [45], and (iv) signal transduction requires only electronic instrumentation, which can also be compact and produced at low costs (e.g., glucometer). Electrochemical methods typically used for bioanalysis can be divided into two large groups: potentiometric and controlled-potential techniques (Figure 2).

Potentiometry operates in chemical equilibrium, where voltage (potential difference) is measured between a reference and a sensing electrode, where the reference electrode provides potential independent of different possible samples, while the sensing electrode potential depends on the concentration of the measured analyte. These electrodes are constructed considering the series of potential steps formed across the different interfaces in the system. Electrical potentials may develop on the surface of the electrode (e.g., chloride (Cl^−^) on silver/silver chloride (Ag/AgCl) or proton (H+) on metal oxide (MOx) surfaces), or across the selectively permeable membranes (ion-selective membranes). An ion-selective electrode (ISE) involves both of these potential steps, first on the membrane separating the sample from the inner filling solution and second on the electrode, which relates it to the potential in a measurement wire. If the inner filling solution (e.g., potassium chloride (KCl)) contains known concentrations of the analyte (e.g., potassium (K^+^)) and ion reacting with the electrode (e.g., Cl^−^ in case of Ag/AgCl electrode), the only variable potential would rise on the membrane due to the change of external analyte concentration, which can be then measured.

**Figure 3:**
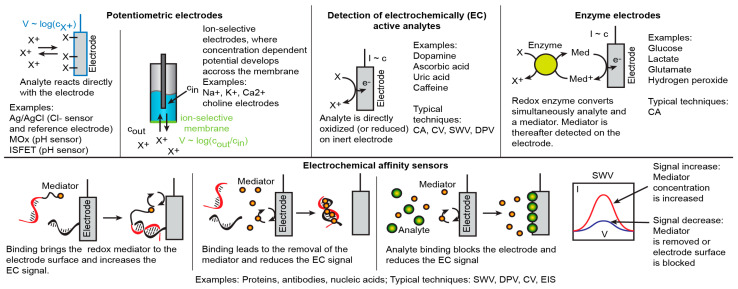
Examples of common electrochemical analysis schemes relevant to the organ chips (Abbreviations: Ag/AgCl Silver/silver chloride, Cl^−^ chloride, MOx metal oxide, ISFET ion-sensitive field-effect transistor, Ca^2+^ calcium, X analyte, Med mediator).

Controlled-potential techniques, on the other hand, assess reaction kinetics and movements of ions. Here, potential between the solution and working electrode surface is controlled by a potentiostat, and the resulting electrical current through the electrode is measured. The current has two contributions, one from the movement of ions (non-Faradaic) and another from the electrochemical redox reactions (Faradaic). A variety of techniques are distinguished based on the potential waveforms used and how results are presented and analysed, each bringing specific advantages, such as response time, resolving reactions by their redox potentials, separating mass-transport and electron transfer or Faradaic and non-Faradaic contributions. Most relevant techniques for biosensing are summarized in Figure 2. Only some analytes are electrochemically (EC) active (e.g., dopamine, ascorbic acid) and can be detected directly on a plain inert electrode; most others, however, require chemical assay schemes to produce electrochemically measurable signals (Figure 3). In case of small molecule metabolites (e.g., glucose, lactate, glutamate), enzyme electrodes can be used, where the corresponding enzyme (e.g., oxidoreductases, hydrolases) reduces or oxidizes the analyte with concurrent redox reaction of a mediator (e.g., ferrocene, methylene blue, quinones). A redox mediator will react then on the electrode to produce electrochemical current. Such electrodes can be well suited for continuous real-time monitoring of cell culture systems. Large biomolecules (e.g., proteins, antibodies, nucleic acids) are mostly detected with affinity sensors based on molecular recognition, which can have numerous effects on EC signals. For example, a binding mediator or redox enzyme on the electrode surface can lead to an increase in the signal, while removal of the mediator (e.g., hybridized DNA binding ruthenium hexamine mediator) or blocking the electrode surface would reduce the corresponding EC current. However, many affinity assays are not directly suitable for real-time continuous monitoring since strong binding might be not reversible. This can be overcome by intermittent electrode regeneration steps [46], where previously bound material is removed, making the electrode surface again available for the analyte. Such regeneration, however, requires more complex reagent handling schemes (e.g., based on microfluidics). In the following paragraphs, we describe recent examples of electrochemical sensors used for cell culture models.

### 3.1. Selectivity of Electrochemical Analytical Methods

Electrochemical techniques can be both highly sensitive and selective. Here, we briefly discuss the limitations of these techniques. Selectivity in potentiometric sensors based on direct analyte reactivity with the electrode (e.g., Cl^−^ in Ag/AgCl) is due to the chemistry of the electrode materials. These electrodes, however, can suffer from cross-reactivity with interfering species. For example, the Ag/AgCl electrode has cross-reactivity with S^2−^ (K~10^15^) SO_4_^2−^ (K~10^−8^), I^−^ (K~10^6^), Br^−^ (K~10^2^), OH^−^ (K~10^−3^) ions, where K value reflects approximate selectivity coefficient over Cl^-^ [47]. As can be seen, electrodes can have much higher sensitivity towards interfering ions compared to the target, therefore requiring careful control of the concentration of the interfering ions in the sample (e.g., Br^−^ is not common in biological samples). Even though Cl^−^ concentration is not highly variable in the context of most in vitro biological models, the Ag/AgCl electrode is highly important as a reference electrode for both potentiometric as well as amperometric and voltametric sensors in many electroanalytical applications in life sciences. In order to achieve better stability as a reference, such electrodes can be isolated by a barrier, which isolates interfering ions. For example. Matsuomto et al. [48] have shown how perfluorocarbon polymer membrane significantly improves the stability of Ag/AgCl reference in physiological conditions. Similarly, selective membranes are also commonly used in ion-selective electrodes (ISEs), such as Na^+^, K^+^ electrodes etc. Such a membrane can be highly specific; for example, for Valinomycin-based K^+^ ISEs, the selectivity can be 10^4^ times higher for K^+^ ion compared to very similar Na^+^ ion [49].

In direct chronoamperometry based sensing, selectivity can be a serious concern, as the method does not pose intrinsically high selectivity. For example, dopamine and epinephrine detection in the presence of ascorbic and uric acids with similar redox potential. However, using nanomaterial electrodes (e.g., with silver nanoparticles—AgNPs), the selectivity can be increased significantly [50] (e.g., over 10^4^× selectivity of dopamine over ascorbic acid). Enzymatic sensors and techniques involving affinity offer higher selectivity due to the high specificity of biomolecular interactions.

### 3.2. Integration of Electrochemical Sensing

One of the advantages of electrochemical sensing is that the required external instrumentation can be compact, versatile, and of low cost. For example, universal open-source potentiostats for assays have been reported, such as USB connected DStat [51] or Bluetooth operated Universal Wireless Electrochemical Detector (UWED) [52], which both have material cost below 100 EUR, and can support most common EC techniques, such as potentiometric measurements, CA, CV, SWV, and DPV. Further miniaturization is possible by direct CMOS integration of control electronics directly with organ chip devices [53].

## 4. Cell Culture Sensing on-Chip

Biosensors for cells have advanced from only detection of specific analytes to real time monitoring and assessment of cell metabolic activities. Optical sensing is one of the commonly used techniques on-chip for real-time monitoring for cells. This optical sensing includes fluorescence microscopy and spectroscopy, chemiluminescence, infrared spectroscopy, and Raman spectroscopy [54]. These techniques provide non-invasive and non-destructive monitoring of the selected variables.

Although optical techniques present good limits of detection (LOD) and do not interfere with the cell activity [55], the integration of optical sensing in microfluidic chips and 3D bioreactors requires additional setting for an optical window to allow the light path [56,57]. Whereas the integration of optical sensors in microfluidic setting is limited, integrated electrochemical sensors facilitate the continuous monitoring of cellular microenvironment. This not only allows monitoring cell viability assays, but also facilitates the monitoring of drug efficacy at very low concentrations [21]. The response times are faster than the ones presented by optical sensors and the side instruments required are simple and portable compared to the ones used in optical techniques [21,52]. Beyond high sensitivity and easy integration in microfluidic systems and bioreactors, electrochemical sensing also offers low-cost manufacturing and integration.

Electrochemical immunosensors use the biorecognition phenomenon to specifically capture the antigen of interest, and an electroactive or enzymatic label to produce an electrical signal measurable by the transducer. In recent years, these electrochemical sensors have been improved, increase their accuracy and sensitivity for a panoply of analytes, including cancer biomarkers [58,59]. With recent advances in the field of microfluidics, many encouraging developments have been made in cell-based biosensors. These sensors can be integrated inside the cell bioreactor in a microfluidic device, and in direct contact with the fluid being tested. Recent trends move towards 3D cell culture systems with integrated electrodes, which can provide detailed information on disease pathogenesis and physiology. The recognition of biomolecules, including antigens, antibodies, and nucleic acids are largely studied through electrochemical detection techniques. Nonetheless, the detection accuracy, precision, and sensitivity of electrochemical sensors depend on charge transfer and stability of biomolecules. Hence, technological advances in the fields of smart nanomaterials and microfluidics have supported the exponential enhancement of sensitivity and stability in the analytical detection of cancer biomarkers [60,61,62]. However, the electrochemical sensing strategies for RNA diagnosis remains a challenging task due to their small dimensions, similar sequences, and fast degradation [63].

These electrochemical immunosensing systems have already been applied to cancer diagnosis. Wei et al. developed the first multiplexing system based on electrochemical detection of two salivary biomarkers (interlukin-8 mRNA and interlukin-8 protein). The electrochemical sensor consisted of an array of 16 gold electrodes coated with probes for each biomarker. Each array corresponded to a three-electrode system supported by a conducting polymer with streptavidin-modified dendrimer nanoparticles to improve the biocompatibility of the sensor. Amperometry was the chosen electrochemical method since it allowed optimal conditions for the monitoring of both biomarkers. The sensitivity and specificity obtained by the electrochemical sensor is approximately 0.90, which is close to what was reported for conventional PCR/ELISA detection tests, and the LOD for interlukin-8 mRNA and interlukin-8 protein was, respectively, 3.9 fM and 7.4 pG/mL. The researchers also concluded that the AUC (area under the curve) was increased for simultaneous detection of both biomarkers, compared to single biomarker detection, which supports that multiplex detection has a higher accuracy than single biomarker assays [64].

Continuing in the field of oral cancer detection, Malhotra et al. developed an electrochemical platform to detect four proteins associated with head and neck squamous cell carcinoma (HNSCC): interleukin 6, interleukin 8, vascular endothelial growth factor (VEGF), and VEGF-C [65]. The electrochemical sensing was formed by an eight-carbon electrode array, an Ag/AgCl reference electrode, and a Pt counter electrode [65,66]. Glutathione-decorated gold nanoparticles were deposited on top of a layer of poly(diallyldimethylammonium chloride) placed onto the carbon electrodes. Proteins were captured through antibodies bonded to magnetic beads coated with horseradish peroxidase (HRP) and then injected inside the microfluidic platform containing the sensor. The enzymatic reaction catalyzed by HRP generated the electrical signal. Normalizing the means for the four proteins data resulted in a sensitivity and specificity of 89% and 98%, respectively. The sensor also presented a good correlation with ELISA assays. The researchers also verified that the detection limits of the developed sensor were in the femtogram range, lower than the detection limits for commercial multiplexing assays with beads [65].

## 5. On-Chip Electrochemical Sensing in 3D Cell Culture

Electrochemical sensing in microfluidic devices has been widely used for development of biosensors/immunosensors [61]. Integrated sensors for the detection of enzymatic immunoassays, and tumour marker analysis using microfluidic systems are new directions in sensorised 3D cell culture models. These sensors display conventional electrode setups and use amperometric, voltammetric, potentiometric, and impedimetic techniques to provide molecular level sensitivity. Electrochemical sensing has also been incorporated in microfluidic systems towards cell counting purposes, mainly applied to the detection of CTCs. Systems for the detection of CTCs have become popular, since their enumeration is relevant for cancer prognosis and their study could improve treatment effectiveness [67,68]. Various techniques including mass spectrometry, electromagnetic spectroscopy, fluorescence, and electrochemistry have been applied for on-chip early detection or capture of cancer cells [39,69,70,71,72]. Modern fluorescence-activated cell sorting machines can optically process large numbers of cells in reduced time, but requiring sophisticated optical instrumentation and analysis software, which are very costly and not possible to transform in easy-to-use diagnostic tools [73]. On the other hand, impedance-based electrochemical methods are much faster and simpler than optical methods, can be integrated in small micro devices close to the cell surfaces, and do not present any optical limitations [74]. The electrochemical detection of cells is non-invasive and provides label-free analysis and characterization, providing information on membrane capacitance, resistance, and cytoplasm conductivity [75].

Different types of 3D model systems are used with electrochemical sensing and provide information with high sensitivity and accuracy. The following sections will discuss recent electrochemical tools implemented in OoC systems for the study of cell migration, drug efficacy, pH and O_2_ detection, and rare cell counting. A summary of all the systems is included in Table 1.

### 5.1. Cell Migration in 3D Cell Culture

**Figure 4:**
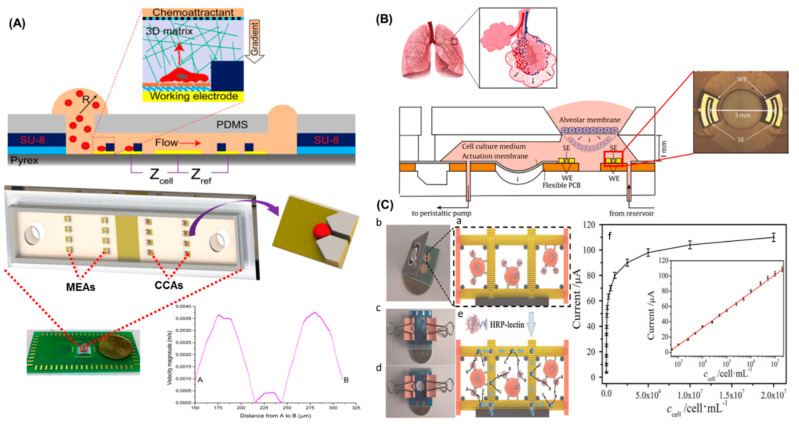
(**A**) Schematic of sensor chip presenting the capture of a single cell and its electrochemical detection. Photography of the sensor unit onto a printed circuit board (PCB), including a magnified image of the microchannel for cell trapping onto the electrodes. The sensor chip consisted of microelectrode arrays (MEAs) and cell capture arrays (CCAs) connected by a microfluidic channel. The inset shows the V-shaped cell trapping structure. The graph shows the flow rate across the system, corresponding to a velocity in the inlet of 2 mm/s. (Copyright) (**B**) Cross-sectional view of the micro-impedance tomography system integrated in the lung-on-chip. The sensing and working electrodes (SE, WE) were placed below the membrane used to support the cell culture. Micro channels permitted the connection to a reservoir for the injection of solutions directly into the basal compartment. The inset shows one of the sensing regions, consisting of two pairs of electrodes separated 3 mm. (Copyright) (**C**) The scheme shows a representative assay in the electrochemical lab-on-paper cyto-device: (a) Immobilisation of horseradish peroxidase (HRP)–lectins onto the cell surface. (b) Folded system. (c) The folded system was clamped in between two circuit boards. (d) Opposite side of (c). (e) Cartoon representing the electrochemical detection of HRP onto the cell surface. (f) Plot of the response against cell concentration, where the inset shows the calibration curve. (Copyright).

Cell migration is a natural process in normal tissues during morphogenesis or even in wound repair. Single cells can migrate, like leucocytes do when our immune system is triggered or collectively as sheets like epithelial cells. Moreover, cell migration is also associated with several diseases, as it happens with tumour formation. Single cancer cells or clusters migrate through the circulatory system, spreading around the entire organism and forming metastases. Therefore, it has become very relevant to study such cell migration process in order to develop better therapeutic strategies [87]. Tumours are 3D structures with a complex microenvironment, so to properly study them and obtain accurate results, it is required to build 3D cell cultures, which mimic the in vivo conditions. Multicellular spheroids are one of the most used 3D culture types and refer to three-dimensional, round shaped cell aggregates consisting of multiple single cells [88]. These structures are easily formed and can often be integrated in microfluidic devices. Tien Anh Nguyen et.al. presented a microfluidic device integrated with impedance sensors to study the movement of individual cancer cells in 3D matrixes (Figure 4A). This system was able to immobilize single cancer cells onto microelectrode arrays prior to in-situ culture and impedance detection. Cell migration inside a Matrigel matrix was followed using MDA-MB-231 cells as a metastatic model, and showed a sudden increase of impedance of about 10 Ω/s. It was also presented that no change in impedance signal was observed using less aggressive MCF-7 cells. This type of 3D sensor chip allowed fast and specific detection of cell migration in a 3D matrix [24].

In another research, Heinz-Georg Jahnke et al. reported on the migration of cells from tumour tissue, with the purpose of studying the metastasis processes. The researchers engineered a novel impedimetric high-dense microelectrode array, and MDA-MB-231 breast cancer cells and two melanoma cell lines were used to recreate viable tumour models. Impedimetric measurements took place for 144 h to assess cell migration and were complemented using optical microscopy and standard transwell experiments. The results of the impedimetric measurements revealed cell proliferative effects. When control spheroids were treated with mitomycin-C, proliferation was inhibited. Thus, this high-density arrays showed the potential of impedimetric monitoring for cell migration analysis in lab-on-a-chip systems [6].

Mermoud, Y. et al. presented a novel lung-on-chip with integrated micro-impedance tomography. This system, which recapitulated the alveolar barrier and included a cyclic mechanical strain to simulate breathing, enabled the detection of resistivity changes in the barrier. The permeabilization of the barrier translated in an impedance decrease of 7% using a lung epithelial cell monolayer. The system was able to simultaneously record impedance, while mimicking respiratory movements, and translated the results into the mechanical strain caused in the alveolar barrier. Researchers also monitored the difference in the mechanical strain between the as-seeded epithelial cells onto the membrane and the confluent monolayer formed after 24 h, resulting on a 0.4% difference. The system used a flexible printed circuit board attached to the lung-on-chip system (Figure 4B), and provided impedance-based monitoring of cells and organs without affecting the biomimetic capabilities of the chip [7].

Min Su et al. developed an electrochemical lab-on-paper cyto-device (ELPCD) for the detection of cancer cells, and the evaluation of glycans in their surface. In this system, the working electrode consisted of a 3D Au-paper modified by aptamers. A sandwich arrangement is used for sensitive and reproducible cell detection using horseradish peroxidase-lectin as the electrochemical probe (Figure 4C). The analytical performance of the system was demonstrated showing a linear response for the detection of K562 cells within the calibrated range, between 550 to 2.0107 cells mL^−1^. Afterwards, the ELPCD was used to determine the presence of multi-glycans on the cell surface and the modification in their expression while applying a drug treatment, with the aim to understand the relevance of glycomics for clinical diagnostics of cancer [89].

### 5.2. Chemical Detection in 3D Cell Culture for Drug Screening

**Figure 5:**
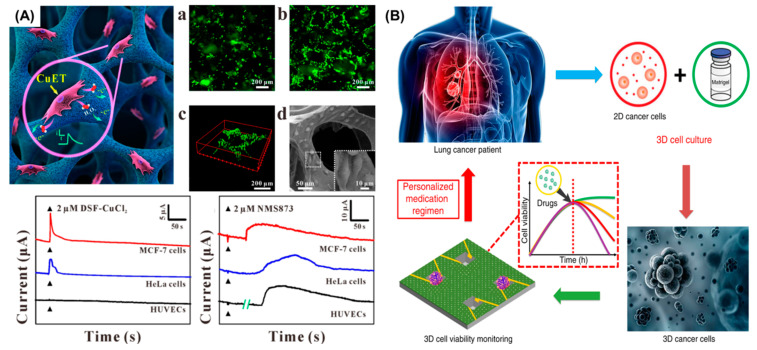
(**A**) Fluorescent micrographs of HeLa cells cultured on polydimethylsiloxane scaffolds coated with poly (3,4-ethylenedioxythiophene) and modified with platinum nanoparticles for (a) 24 h and (b) 72 h (c) 3D reconstruction of confocal images of HeLa cells cultured on the composites for 7 days. (d) Scanning Electron Microscopy images of cells cultured onto the composites for 24 h. The inset displays magnified cells attached onto the scaffold. The graphs at the bottom show the amperometric response of cells cultured onto the composite for 5 h under (a) 2 μM DSF-CuCl2 and (b) 2 μM NMS873 stimulation. (Copyright) (**B**) Schematic of the 3D ECIS for antineoplastic drug screening (Copyright).

The current standard process for drug screening is split into three stages: 2D cell culture-based tests, animal model, and clinical trials. However, only approximately 10% of the drugs set for testing pass all the procedures and are able to be approved and reach the market [90]. Such low rates are mainly attributed to unrealistic environment represented by 2D cell cultures. A 3D structure makes cells less sensitive to drugs compared with cells in a 2D monolayer, which can be due to receptors distribution in cells membrane or even the fact that cells in 3D structures tend to be in different stages of development. Zhang et al. reported on a polymer-based scaffold for 3D cell culture based on conductive polymer coatings. In this case, the polydimethylsiloxane scaffold was coated using poly (3,4-ethylenedioxythiophene) and later altered adding platinum nanoparticles. The 3D system showed desired biocompatibility for long-term cell culture, as well as outstanding electrocatalytic activity for sensing (Figure 5A). The system demonstrated real-time measurement of reactive oxygen species as released from cancer cells when treated using an anticancer drug, showing its potential to monitor cancer treatment [40]. Torisawa et al. developed a silicon chip with an array of cell panels integrated with multi-channel drug containers. Human breast cell line (MCF-7) was cultured in collagen inside the cell panels. Given the chip structure, the effects of three anticancer drugs were able to be simultaneously detected by using the SECM technique to measure the respiratory activity of cells. A comparison of cell proliferation and chemosensitivity in 2D and 3D culture was also performed. The researchers concluded that each cell panel in their chip independently measured different stimuli using only a volume of 22 µL of drug [91]. In a recent research, Yuxiang Pan et al. developed a 3D microgroove impedance sensor (MGIS) for antineoplastic drug testing. To validate and prove the efficiency of the sensor, the researchers used a 3D lung cancer model combined with the MGIS sensor. 3D cultures were made using A549 cells, which were encapsulated inside a Matrigel (Figure 5B), and cell viability was measured using EC impedance spectroscopy technique (EIS). Drug efficiency was tested using antitumour drug cisplatin in the following concentrations: 10, 100, and 1000 μM, and the drug synergy effect was also explored in the drug assays adding concentrations of gemcitabine/pemetrexed along with cisplatin. For further analysis, the investigators decided to establish a comparison between results obtained using the MGIS chip and a 2D biosensor, detecting significant differences in the drug responses between an in vivo like environment and 2D cells assays [76].

### 5.3. Physical Detection in 3D Cell Culture

#### 5.3.1. pH Detection

**Figure 6:**
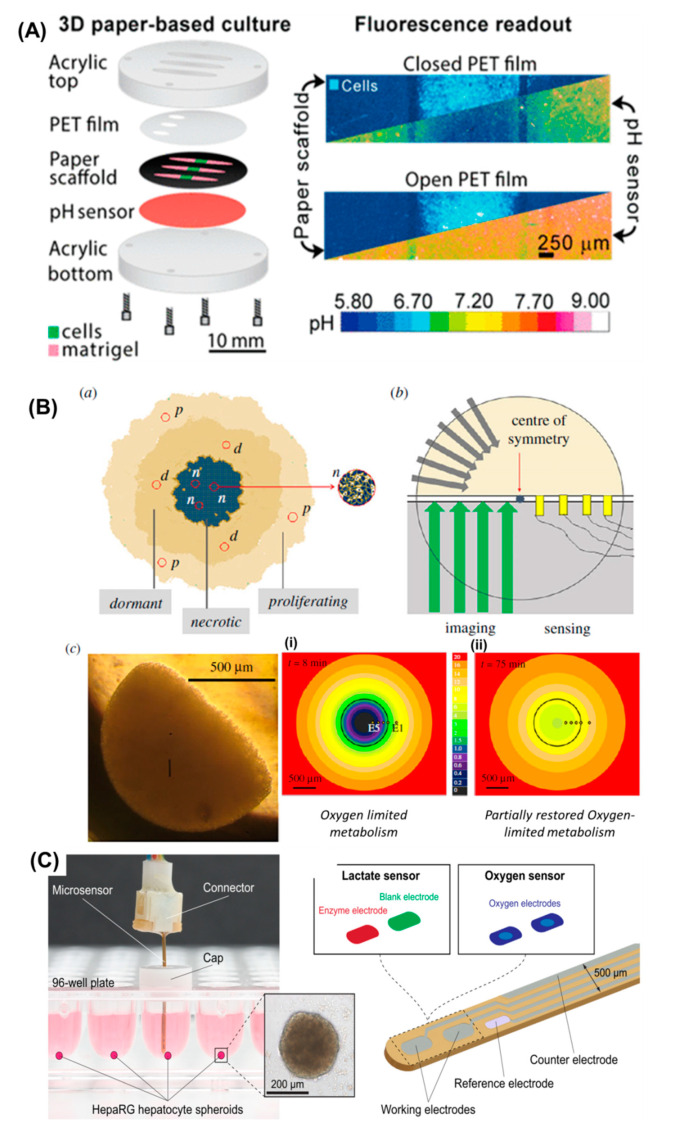
(**A**) Schematics of Optode to produce pH maps in 3D cell cultures based on paper (Copyright) (PET—Polyethylene terephthalate). (**B**) (a) the scheme shows the inside of a 3D multicellular tumour spheroid consisting of thousands of cells. Cells at the spheroid surface have access to oxygen and nutrients, while being able to clear metabolic waste during proliferation. The center is composed of a necrotic core where nutrients and oxygen are limited and waste products are accumulated, hence producing acidification. Dormant cells (viable, but inactive) accumulate between the proliferating and necrotic regions. (b) In a model spheroid, the entry of nutrients and oxygen (grey arrows) is maintained. (c) The image shows a model of drug-resistant breast cancer cells cultured for four days into a hemi-spheroid. Oxygen maps are obtained in and out of the spheroid (the column shows the colour calibration); (i) maps are obtained after 8 min of placing the spheroid onto the electrode array, when the highest oxygen consumption is found. (ii) Oxygen metabolism is restored in part after adding 2,4-dinitrophenol at minute 75 (Copyright). (**C**) A sensing system is incorporated in a multi-well-plate. The scheme shows the electrode layout in the sensing tip. The lactate sensor uses an enzyme immobilised in the working electrode and a diffusion limiting membrane for the continuous analysis of metabolic activity for three days (Copyright).

Acidification of microenvironment is important in stimulating the aggressive cancer phenotypes and, as such, pH monitoring is particularly relevant in cancer. The extracellular pH values for tumour tissues are more acidic than in normal tissues, falling in a range of 6.4–7.0, while the pH in healthy tissue is neutral or slightly alkaline [92]. This is generally attributed to the Warburg effect, which consists in using anaerobic glycolysis over oxidative phosphorylation even in the presence of oxygen, by cancer cells [93]. The excess of lactate is considered the cause for the acidification of the extracellular environment. Low extracellular pH values characterize the tumour microenvironment and its manipulation may help in the development of new therapeutic strategies, enhancing, therefore, the importance of studying this parameter [92]. Optical sensing has been vastly applied for pH monitoring, unlike electrochemical sensing. However, and due to the advantages of electrochemistry, in the last years, that trend has started to switch. Parmiss Mojir Shaibani et al. used a light-addressable potentiometric sensor incorporated into a pH-sensitive nanofiber-based hydrogel to monitor in real time the extracellular acidity of MDA-MB-231 cancer cells by detecting pH changes in the media. The hydrogel nanofiber swelling caused change in the photocurrent signal, which reflected a pH change. All the measurements were done using a linear sweep voltammetry strategy and a sensitivity of 74 mV/pH was obtained. Moreover, a drug called doxorubicin was used in cancer cells (MDA-MB-435MDR) to study its effects on pH. A decrease in pH was confirmed when cells were treated with the drug; however, when coupled with metabolic enzymes inhibitors, a decrease in acidification was observed [86]. Rachael M. Kenney et al. developed paper-based tumour models and methodology to understand how cells respond to acidic conditions. They described pH-sensing optodes that were able to obtain maps of pH gradients with high spatial and temporal resolution in a paper-based culture system. The sensor was created by incorporating microparticles containing pH-sensitive and pH-insensitive dyes into a polyurethane hydrogel, and then coated with a transparent substrate (Figure 6A). The described films presented a quick response time and stability in cell cultures, with no toxicity effects. The films are sensitive in the relevant pH range to distinguish normal and tumoural tissues [94].

#### 5.3.2. Oxygen Detection

Monitoring oxygen (O2) levels has major importance when it comes to studying tumour tissues. With tumour growth, an oxygen concentration gradient is created, and some regions become hypoxic due to the chaotic and insufficient blood supply. This natural gradient is also influenced by the diffusion of molecules through the ECM and it generates layers where cells are in their proliferating state, quiescent phase of their cycle, or in a necrotic state [34]. Blood capillaries distribute nutrients and oxygen to neighboring tissues, while transporting metabolic waste. Dishn B. Sheth and co-workers obtained dynamic oxygen maps inside 3D tumour hemispheroids by means of a non-invasive microelectrode array. This strategy provided access to otherwise less-accessible oxygen levels inside the spheroid, by providing high throughput measurements and with high potential in the study of cancer cell biology, drug discovery, and personalized medicine. They demonstrated how hypoxia influences cancer treatment by obtaining oxygen distributions inside a 3D tumour hemispheroid, using their microelectrode array (Figure 6B). For this purpose, breast cancer cells were used and kept with agarose into a spherical shape. The oxygen levels were measured using differential linear scan voltammetry. To confirm the viability of cultured cells inside the spheroid, metabolic modulators were also added during the real-time monitoring of O_2_ concentrations. [95]. James Jenkins et al. presented a model based on hybrid microporous scaffolds made in polystyrene and loaded with O_2._–sensitive phosphorescent dyes to measure O_2_ distribution in living cells and compatible with fluorescence microscopy. These scaffolds presented a good correlation between phosphorescence intensity and O_2_ concentration. They were also suitable for long time cell culture, maintaining unchanged cell viability. Further, such scaffolds were tested with cancer cells, cellular aggregates, and tissue slices, showing their capability to inform oxygen levels at different measurement depths and in various cell densities, and correlated with changes in respiration activity, viability, and in response to incubation with drugs. With the same methodology, researchers presented multiplexed sensing and decreased O_2_ in the scaffold. The O_2_-sensitive scaffolds have the potential to provide improved control conditions for 3D tissue cultures, with various applications in cancer diagnostics and treatment selection. [96]. In a different work, Andreas Weltin and coworkers used electrochemical microsensors to measure metabolic activity from hepatocyte spheroids. The system allowed the detection of oxygen and lactate, while integrated in a multi-well-plate to allow continuous long-term monitoring of metabolites in a precise manner (Figure 6C). The limit of detection for lactate in cell culture medium was 50 μM and production rates went up to 5 μMh^−1^ over three days. In the same experiment, oxygen levels did not suffer any alteration in the well. The capacity to assess metabolic production in a 3D culture using standard tools provided an advantage for the analysis of in vitro toxicology [78].

### 5.4. Rare Cell Detection

It is also very important to develop a methodology to detect CTCs with high efficiency. The isolation strategies for single cell remains a challenge in case of CTCs and disseminated tumour cells (DTCs) because of their low concentration in the sample of bone marrow or blood, requiring sample pre-enrichment prior to sensitive detection. Li An and coworkers used a gold-plated polymeric substrate modified with benzoboric acid to enrich cancer cells from clinical samples. This approach provided the advantage of capturing CTCs directly onto the electrode surface for cell enumeration. The results show that this system was able to isolate down to five cancer cells per mL of sample while avoiding pre-processing [97]. Loc Quang Do and coworkers developed a differential technique incorporated into a sensing structure to reduce signal to noise for the recognition of ultralow signals. This microfluidic platform employed dielectrophoresis manipulation with an integrated sensitive and selective capacitive biosensor for rare biological cell detection. In this system, dielectrophoresis forces were created through a sinusoidal signal of 16 V_pp_ and a frequency of 1 MHz and applied on a suspension of target cells contained in the chamber. The measurements were carried out at 300 kHz to detect S-180 cultured cells. The sensitivity of 3 mV/cell was obtained for the devices [98]. Mojgan Ahmadzadeh Raji and coworkers reported on the design of an aptasensor towards colon cancer detection. They demonstrated aptamer capacity to recognize cells of interest using fluorescence microscopy, flow cytometry, and electrochemical experiments. HCT116 and HT29 cell lines were selected as colon cancer models, while HEp-2 cells were used as a negative control. Finally, the electrode was functionalized to immobilize the aptamer, and the sensor demonstrated a linear response for cell enumeration [99].

## 6. Conclusions

Over the last few years, EC sensors have been integrated in OoC models to allow real-time monitoring of cell migration, pH and O_2_, as well as for the detection of rare cells and even for drug screening applications. The future directions for sensorised 3D cell culture systems based on electrochemistry are focused on the development of new functional polymer hydrogels and microfabrication techniques. For example, one-step electrospinning techniques enable functional entities to be presented on nanofiber surfaces during the process to fabricate scaffolds. Therefore, electrodes presented in nanofiber form in the scaffold can interface directly with the cells growing on its surfaces. However, the main challenge for these functional biomaterials is to meet the stringent requirements for cell and biological compatibility. On the other hand, biosensing platforms integrated with electrochemical electrodes to isolate and detect cancer cells from biological fluids have been reported to be extremely effective and able to address challenges and requirements related with this complex task. Sensing elements incorporated in 2D cultures and 3D scaffolds have been successfully realized, offering electrochemical ways for improving micro-scale understanding of cell culture microenvironment, and will facilitate advanced organ-on-chip studies in the future.

The trends in this field are clearly moving towards increasing abilities for the detection and development of precision clinical testing devices that can be personalised for efficient cancer diagnosis and treatment selection. Electrochemical transduction strategies stand as a competitive candidate for reliable label-free and multi-analyte detection in the future. However, selectivity and quantitative evaluation is still an unsolved puzzle, which needs to be ensured to be able to provide the high standards needed by the clinical diagnostic sector.

## Figures and Tables

**Table 1:** Summary of electrochemical sensors incorporated in 3D cell culture systems and their application.

Cell Culture Type	Application	Electrochemical Sensing Method	Limit of Detection	Reference
3D cancer cell model: A549 cells cultured in a Matrigel	Development of a multidimensional microgroove impedance sensor (MGIS) for the real-time analysis of cell viability, for drug sensitivity testing in 3D cancer models	3D ECIS (electrochemicalimpedance spectroscopy)	10 μM	[76]
A549 lung cancer cells cultured in several types of sol-gels (alginate, collagen, matrigel)	Development of a electrochemical biosensor for cytotoxicity assay on 3D cell culture	SWV (Square Wave Voltammetry)	-	[77]
Single human HepaRG hepatocyte spheroids	Development of an electrochemical microsensor system integrated into 3D cell culture environment, to monitor online lactate production and oxygen consumption	Chronoamperometry and Amperometry	lactate sensitivity 5 μM to 30 μM	[78]
3-aminophenylboronic acid (APBA) functionalized graphene foam (GF) network cultured with HeLa cells	APBA-functionalized GF networks for cell culture and electrochemical sensing, to monitor in real time gaseous messengers H_2_S	CV (Cyclic Voltammetry) and Amperometry	50 nM	[79]
PEDOT-coated PDMS scaffold followed by platinum nanoparticles (Pt-NPs) electrodeposition cultured with HeLa, MCF-7 and HUVECs cells	Development of a novel 3D electrochemical sensor, used to monitor in real time the release of ROS, induced by a new anticancer drug	CV (Cyclic Voltammetry) and Amperometry	76 nM	[40]
3D lung cancer spheroid models (A549, H1299, H460)	Drug testing in lung cancer spheroids using interdigitated electrodes	Electric impedance	-	[41]
nano-Mn_3_(PO4)_2_—chitosan cultured with 4T1 cells	Screen printed CTS-Mn_3_(PO4)_2_ electrodes for the detection of superoxide anions released by cells in a 3D cell culture model	CV (Cyclic voltammetry) and Chronoamperometry	9.7 nM	[80]
SK-BR-3 cells inserted in a 3D electrochemical system, mimicking the in vivo microenvironment	Paper electrode with platinum nanospheres to capture cancer cells and determine in real-time the H_2_O_2_ released from cells	Electrochemical impedance spectra (EIS)	0.0001 μM	[81]
HepaRG human hepatocyte spheroids	Development of an electrochemical monitoring platform, for the monitoring of lactate production rates	Amperometry	1 μMh^−1^	[82]
Dipeptide-derived hydrogel matrix cultured with HeLa cells	CSH-hydrogel that electrochemically monitors superoxide anions release	(CV) Cyclic voltammetry and Amperometry	0.34 nM (with cells) and 0.35 nM (without cells)	[83]
Human hepatocyte spheroids	Electrochemical immunosensor integrated in a microfluidic perfused liver bioreactor for in-line monitoring of cell-secreted biomarkers.	Amperometry	0.03 ng/mL (Transferrin)	[7]
Liver and heart on-a-chip models	Multi-organ on-a-chip platform with a microfluidic breadboard, controlled by pneumatic valves, and integrated with physical, biochemical, and optical sensors, for real time analysis of cell micro-environment	EIS (Electrochemical Impedance Spectroscopy)	albumin: 0.09 ng/mL; GST-α: 0.01 ng/mL; CK-MB: 0.0024 ng/mL	[46]
CDs@ZrHf-MOF-based (bimetallic ZrHf-MOF coupling with CDs) aptasensor used as scaffold to detect HER2 in breast cancer cells	Scaffolds of CDs@ZrHf-MOF are used to anchor aptamers specific to determine human epidermal growth factor receptor-2 (HER2) in living MCF-7 cells.	EIS (Electrochemical Impedance Spectra)	19 fg/mL for HER2	[84]
Electrochemical microfluidic paper-based cyto-device to detect HL-60 cells	Microfluidic paper-based electrochemical cyto-device for cancer cell detection and in situ screening of anticancer drugs	DPV (Differential Pulse Voltammetry)	350 cells/mL	[85]
pH sensitive hydrogel nanofiber	Light Addressable Potentiometric Sensor integrated with pH sensitive hydrogel nanofibers (NF-LAPS) to measure pH changes in breast cancer cell lines	LSV (Linear Sweep Voltammetry)	10^3^ mL^−1^	[86]

Abbreviations (alphabetically): CDs—Carbon dots, CSH—Chiral Supramolecular Hydrogel, HeLa—Henrietta Lacks (cell line), HepaRG—human hepatocytes (cell line), HL—human leukemia (cell line), HUVEC—Human umbilical vein endothelial cells, MCF- Michigan Cancer Foundation (cell line), MOF—Metal-organic framework, PDMS—polydimethylsiloxane, PEDOT—poly(3,4-ethylenedioxythiophene), SK-BR- Sloan-Kettering Breast cancer (cell line).
